# Analysis of the bony geometry of the acromio-clavicular joint

**DOI:** 10.1186/s40001-018-0348-3

**Published:** 2018-10-23

**Authors:** Moritz Crönlein, Lukas Postl, Marc Beirer, Dominik Pförringer, Jennifer Lang, Frederik Greve, Michael Müller, Peter Biberthaler, Chlodwig Kirchhoff

**Affiliations:** Department of Trauma Surgery, Klinikum rechts der Isar, Technical University of Munich, Germany, Ismaninger Strasse 22, 81675 Munich, Germany

**Keywords:** AC joint configuration, AC joint injuries, Acromioclavicular joint dislocation, Lateral clavicle fractures

## Abstract

**Background:**

The primary goal of this study was to analyse the anatomic configuration of the acromio-clavicular joint in a healthy population to be able to develop a classification in a second step. On the basis of the primary findings a secondary goal was to find potential clinical indications in refer to AC-joint dislocation and lateral clavicle fractures.

**Methods:**

The upper thoracic aperture including both shoulder joints as well as both sterno-clavicular joints was retrospectively reformatted in a bone kernel in axial orientation with 0.6 mm slice thickness out of existing multiple trauma or post mortem computed tomography (CT) scans. The DICOM data was converted into the STL file format using a three dimensional (3D) reconstruction software (Smartbrush, Brainlab, Feldkirchen, Germany). The data analysis was performed using a 3D—Computer Aided Detection (CAD) Software (BioCAD, Technical University Munich, Germany). For the analysis, the angle between the cranial surface of the acromion and the tangent to its articular surface was evaluated. Accordingly, the angle between the cranial surface of the clavicle and the tangent to its articular surface was assessed.

**Results:**

Overall CT-datasets of 80 healthy patients (40 males, 40 females, mean age 45 ± 8 years) were enrolled and evaluated regarding the configuration of the AC-joint. In this context, three statistically significant (*p* < 0.001) different configurations of the AC-joint in terms of overhanging acromion, neutral type, overhanging clavicle were identified. The “overhanging acromion” type of AC-joint configuration turned out to be the most common type (46.2%) followed by the “neutral type” (38.4%) and finally the “overhanging clavicle type” (15.4%).

**Conclusions:**

We assume that the shown differences of the AC joint congruency might play an important role in the development of different shoulder injuries resulting from the similar trauma mechanism. However, the proof of these assumptions will be the focus of future studies.

## Background

The acromioclavicular (AC) joint is very commonly affected by injuries in terms of direct or indirect trauma as well as by degenerative changes. To be able to treat AC joint pathologies of any kind, physicians need a good understanding of the anatomy and mechanics of the AC joint. In the recent past, significant effort has been made analyzing the ligamentous stabilizers of the AC joint [1–3]. Especially the coraco-clavicular (CC) ligament, composed of the conoid and trapezoid ligament, presents a highly valuable passive stabilizer notably for vertical translation [4].

Regarding the bony anatomy, the clavicle is known for its double curved configuration. Its lateral end is flattened and presents with prominent landmarks on its inferior surface. From the anatomical point of view the acromion articulates with the lateral clavicle via the medial facet with its orientation posteriorly and laterally whereas the acromial articular surface is oriented medially and anteriorly [5]. The average size of the AC joint surface was described as 9 mm in vertical by 19 mm in antero-posterior orientation [6, 7]. The current literature provides sufficient information regarding the rather complex anatomy of the double curved clavicle as it is described that on average of 40 mm medial of the AC joint the clavicle presents an anterior sweep with an increased thickness of the superior cortical bone ranging between 1 and 4.5 mm [2, 8, 9].

Knowing about the numerous studies of the anatomy of the AC region [1, 2, 5, 8–10], the aim of our study was to evaluate the configuration of the AC joint on the basis of CT Data sets of a young population without pre-existing joint pathologies.

## Methods

### Subjects

Patients who underwent a computed tomography (CT) either in terms of multiple trauma CT scan or post mortem CT were enrolled. Bony pathologies of the upper extremities, anamnestic prior shoulder surgery (available medical records, scars or third-party medical history) and signs of significant AC joint arthrosis were considered as exclusion criteria. The study was approved by the Institutional Review Board (IRB approval).

### Data acquisition and analyzing

Computed tomography scans either in terms of multiple trauma or post mortem scans were performed on a GE LightSpeed VCT XTe CT scanner using the following standard examination parameters: 140 kV, 40 mAs, collimation 0.6 mm, pitch 0.75 mm. After rule out of the exclusion criteria on the localizer the CT raw data of the upper thoracic aperture including both shoulder joints as well as both sterno-clavicular joints was reformatted in a bony kernel in 0.6 mm thick axial slices. The resulting DICOM data were converted into a STL file format using a three-dimensional (3D) reconstruction software (Smartbrush, Brainlab, Feldkirchen, Germany). The data were analyzed using a three-dimensional (3D)—Computer Aided Diagnosis (CAD) Software (BioCAD, Technical University Munich, Germany). The cranial surface of the acromion, the articular surface of the acromion, the articular surface of the clavicle and the cranial surface of the clavicle were determined by the software’s best-fit surface equalizing algorithm. The angle between the cranial tangent of the acromion and the tangent to its articular surface was evaluated. Accordingly, the angle between the cranial tangent of the clavicle and the tangent to its articular surface was identified. Analysis was performed in accordance to the description of measuring the lateral distal femur angle (ldfa) [11–13].

Data are provided as arithmetic mean and standard deviation. Statistical analysis was performed using the Sigma Stat 3.1 software (Systat Inc, Chicago, IL, USA) with a level of significance *p* < 0.001.

## Results

CT-scans of the upper thoracic aperture of 80 patients (40 male, 40 female) with a mean age of 45 ± 8 years were enrolled. 60 CT-scans were multiple trauma CT scans and 20 were post mortem CT-scans.

**Fig. 1:**
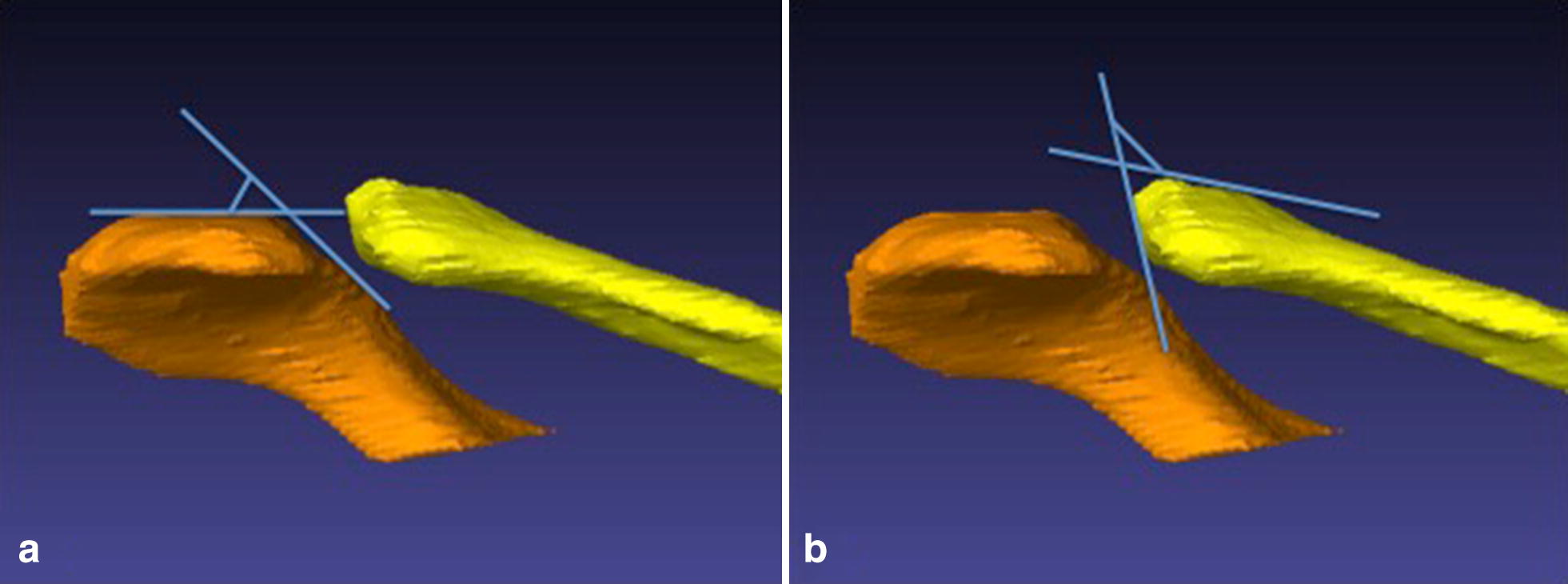
3D illustration of the acromion angle and the clavicle angle. The acromion angle describes the angle between the cranial surface of the acromion and the tangent to its articular surface (**a**), the clavicle angle describes the corresponding angle between the cranial surface of the clavicle and the tangent to its articular surface (**b**)

Both AC joints of each CT scan of the upper thoracic aperture were evaluated, so that in total 160 AC joints were analysed for the anatomic configuration and possible differences (Fig. 1).

**Fig. 2:**
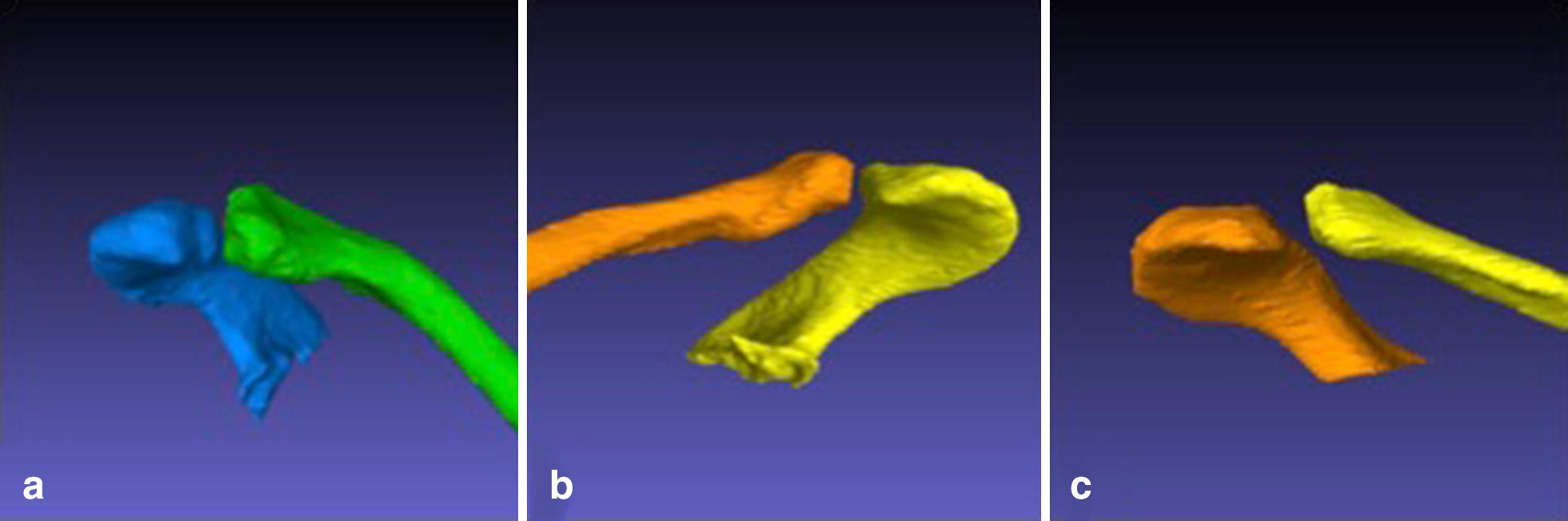
3D illustration of the three different configurations of the AC joint. The three different AC joint configurations are illustrated in this figure: overhanging acromion (**a**), neutral shaped (**b**) and overhanging clavicle (**c**)

Three significantly (*p* < 0.001) different groups could be identified (see Table 1). With 46.2% (*n* = 74) of all examinations the AC joint configuration with an overhanging acromion was determined to be the most common (see Fig. 2a). Besides the neutral shaped type (see Fig. 2b) (*n* = 61) with 38.4%, the overhanging clavicle type (*n* = 45) (see Fig. 2c) with 15.4% of all examinations was determined to be the rarest.

**Table 1:** AC joint configuration

	Type	Incidence (%)	Acromion angle	Clavicle angle
Group I	Overhanging acromion	46.2	112.7 + 7.4°	72.7 + 7.5°
Group II	Neutral shaped	38.4	88.6 + 6.2°	92.9 + 13.7°
Group III	Overhanging clavicle	15.4	71.3 + 7.5°	108.2 + 12.9°

The corresponding angles to the different AC joint types are illustrated in this table besides their frequency distribution. Data are given as arithmetic mean and standard deviation

The summarized angle acromion + right clavicle was determined with 182.8 ± 10.6°, the summarized angle acromion + left clavicle was determined with 183.2 ± 8.8°.

## Discussion

Injuries to the AC joint most commonly result from a direct impact to the shoulder. Although, other possible underlying trauma mechanisms are frequently discussed in current literature [2, 14, 15], only few studies exist, dealing with the influence of the bony joint configuration on the injury pattern [10, 16, 17].

The anatomical and biomechanical analysis of the AC joint resulted in a description of dynamic as well as static stabilizers, with the deltoid muscle and trapezius muscle as primary dynamic and the acromioclavicular (AC) and coracoclavicular (CC) ligaments as primary static stabilizers [6, 18]. Relating to the double curved clavicle, the trapezoid ridge extends anteriorly and laterally across the inferior surface of the lateral third of the clavicle, representing the landmarks of the insertions of the corresponding ligaments [6, 18]. The CC ligament, consisting of the conoid and trapezoid ligament, is known to provide vertical stability [6], whereas horizontal stability is mainly provided by the AC ligament, respectively [14, 19].

On the basis of 41 cases, Urist et al. is known to be one of the first to describe the anatomical variants of the joint structure [10]. He supposed that the different joint configurations might have an influence on the success or failure of AC joint injuries treated in a conservative manner. Although his assumptions might be true, there is still no general concern of the right treatment of AC joint injuries nowadays [20–22]. Consistent with Urist et al. [10], we were able to identify three major types of AC joint configurations on the basis of CT-data of a healthy population. Our findings support the anatomical variants described by Colegate-Stone et al. [17], although they were based on a much older patient group (mean age 81.2 years) [17]. The overlapping acromion considered as type I presented the most common AC joint configuration (*n* = 74, 46.2%). The correspondingly measured mean acromion angle accounted for 112.7°. This AC joint type forms a congruency of the joint resulting in a certain projection towards a superior dislocation of the clavicle. Patients with this AC joint type might rather suffer from lateral clavicle fractures than from AC joint dislocation following a direct impact onto the clavicle resulting in superior dislocation of the clavicle. In contrast, a fall on the outstretched arm, considered as an indirect trauma, drives the humeral head in a superior direction into the acromion with a consecutive superior dislocation of the acromion, while the clavicle remains in the same position [4, 14]. In these cases, patients with an overlapping acromion type would rather suffer from AC joint dislocations than lateral clavicle fractures.

The second most common type of AC joint configuration (*n* = 61, 38.4%), presenting a neutrally shaped joint configuration, presumably does neither promote nor protect from any injuries due to its neutral position of acromion and clavicle and the corresponding angles.

Regarding the third different type of AC joint configuration (*n* = 45, 15.4%) considered as the overhanging clavicle type with a mean acromion angle of 71.3°, the acromion is possibly protected against superior dislocation. Considering this, one might assume that the overhanging clavicle type might promote an AC joint dislocation in direct trauma and a lateral clavicle fracture in indirect trauma contrary to the overhanging acromion type I AC joint configuration.

However, not only the osseous configuration is responsible for the origin of lateral shoulder injuries resulting from direct or indirect trauma. Especially concerning the fact, that osseous differences might be equalized by the cartilage surface or the discus. Rather multiple factors like the already mentioned dynamic and static joint stabilizers, the force of the impact or the bone quality play an important role as well and need to be carefully considered in this context [18, 23].

## Conclusion

Our results show, that the variety of AC joint configurations can be summarized into three different major types: the overhanging acromion, the neutral shaped and the overhanging clavicle type. We assume, that the shown differences in the AC joint congruency play an important role in the development of various shoulder injuries resulting from the same trauma mechanism. To proof our assumptions, further studies are needed.

## Abbreviations

AC joint: acromioclavicular joint
3D: three-dimensional
AC ligament: acromioclavicular ligament
CC ligament: coracoclavicular ligament
ldfa: lateral distal femur angle
